# Oriented growth of porphyrin-based molecular wires on ionic crystals analysed by nc-AFM

**DOI:** 10.3762/bjnano.2.4

**Published:** 2011-01-13

**Authors:** Thilo Glatzel, Lars Zimmerli, Shigeki Kawai, Ernst Meyer, Leslie-Anne Fendt, Francois Diederich

**Affiliations:** 1 Department of Physics, University of Basel, Klingelbergstrasse 82, 4056 Basel, Switzerland; 2 Laboratory of Organic Chemistry, ETH Zurich, Wolfgang-Pauli-Str. 10, 8093 Zurich, Switzerland

**Keywords:** directed growth, KBr, molecular wires, NaCl, nc-AFM, porphyrin, self assembly

## Abstract

The growth of molecular assemblies at room temperature on insulating surfaces is one of the main goals in the field of molecular electronics. Recently, the directed growth of porphyrin-based molecular wires on KBr(001) was presented. The molecule–surface interaction associated with a strong dipole moment of the molecules was sufficient to bind them to the surface; while a stabilization of the molecular assemblies was reached due to the intermolecular interaction by π–π binding. Here, we show that the atomic structure of the substrate can control the direction of the wires and consequently, complex molecular assemblies can be formed. The electronic decoupling of the molecules by one or two monolayers of KBr from the Cu(111) substrate is found to be insufficient to enable comparable growth conditions to bulk ionic materials.

## Introduction

One of the main challenges of artificial photosynthesis and molecular electronics is the controlled growth of molecules on the nanometer scale in a certain direction. For the construction of electronic devices, nanowires are essential components which provide an efficient transport of electrons and/or excitons along specific directions. Compared to semiconductor based devices, self-assembled molecules provide some distinct advantages such as self-healing [1] and a decreased number of defects [2–4]. For some time, studies on molecular growth were limited to metal substrates analyzed by scanning tunneling microscopy (STM) (for a few selected examples see [5–13]). Alkali halide thin insulating films on metal surfaces are often regarded as the model system for both testing experimental methodologies and validating new theories. In particular NaCl thin films have already proved their importance as homogeneous ultrathin spacer layers to separate single molecules from the metal substrate [14–16]. Nevertheless, complete electrical decoupling of such devices from the substrate requires bulk insulators or thick insulating films. Several studies by non-contact atomic force microscopy (nc-AFM) were done on ionic crystals with adsorbed PTCDA [17–22], PTCDI [23] or C_60_ [24]. In the case of porphyrins, the growth [25–27] and electronic properties [28] of stable, monolayered molecular wires on KBr(001) with a length of up to several hundred nanometers have been observed at room temperature (rt). Even the contacting of self-ordering molecular wires by nanolithography was shown recently [29].

Controlled growth procedures of molecules on insulators are often hindered by the weak, unspecific interaction between the molecules and the insulating surfaces which leads to diffusion on the surfaces and assembly of disordered aggregates. One possibility to overcome this barrier is the use of a specific end group which induces an adequate directed dipole moment within the molecule [26,30]. Moreover, high resolution measurements of molecules on insulating surfaces were scarce due to a lack of suitable imaging techniques. However, recent progress in high resolution nc-AFM has given the opportunity to verify the proposed concept of directed growth of molecular wires on insulators [31–33]. Alkali halides offer some distinct advantages compared to other surfaces. Flat surfaces with monoatomic steps and large terraces are easily prepared and electron bombardment leads to well-structured surfaces [34]. Additionally, these materials have rather large unit cells which allow to obtain atomic resolution fairly easily [35–36].

In the work presented here we focus on the influence of the substrate on the growth process of *meso*-(4-cyanophenyl)-substituted Zn(II) porphyrin molecular wires self-assembled on KBr(001) and NaCl(001) studied by nc-AFM. We found that the lattice spacing of the ionic crystal has a direct impact on the growth direction of the wires. Extending the studies, the self-assemblies of molecules onto thin ionic films deposited on Cu(111) revealed that the growth process is also strongly influenced by the metal substrate even for several monolayer of KBr which also indicates an imperfect electronic decoupling.

## Results and Discussion

**Figure 1:**
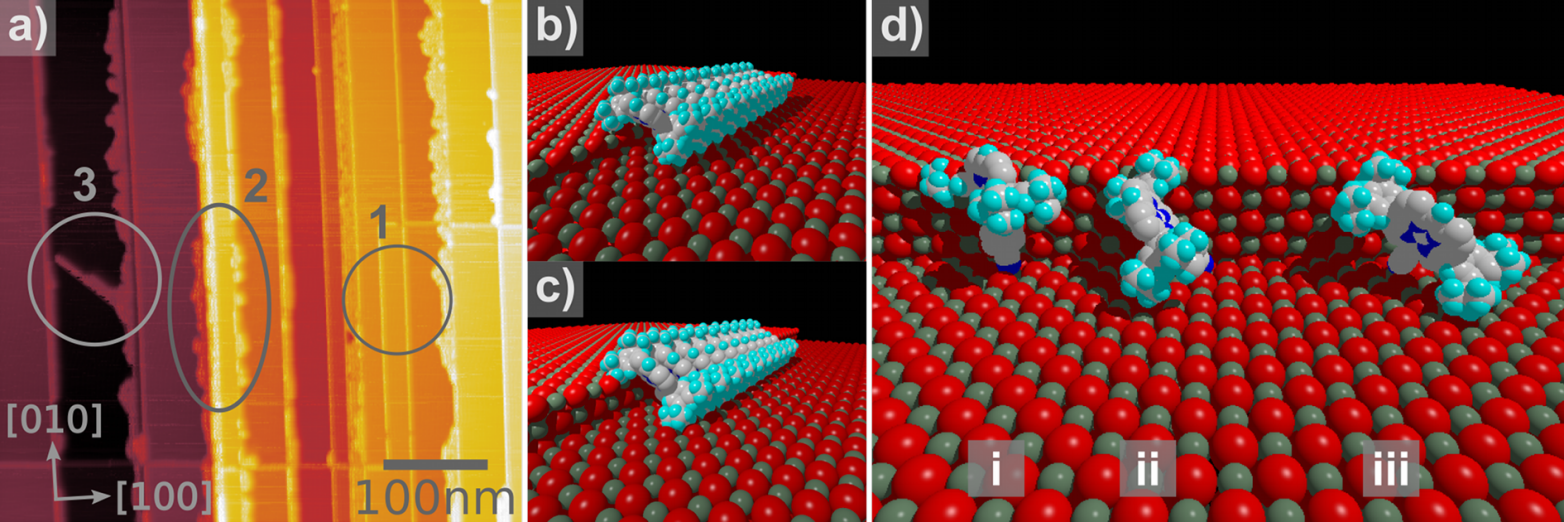
(a) Topographical measurement of molecular structures at KBr step edges showing monowires (1), unordered agglomerates (2) and multiwires (3). Scan range = 500 × 500 nm^2^, *A*_1st_ = 20 Hz, Δ*f*_1st_ = −8 Hz. The arrangements estimated from height profiles along single and double steps are shown in (b) and (c), respectively. The tilt angle of the molecules has to adopt to fulfil geometrical conditions. Along a triple step, one dimensional wires were never observed. (d) Three different orientations of molecules at those edges. The leftmost molecule (i) in (d) is turned by 45° so that the core is oriented along the [110] direction. This orientation does not allow π–π stacking along the direction of the step edges. The molecule in the middle (ii) is tilted by 45° to the surface, making it fit geometrically to the step. (iii) combines those two angles and could be considered as a possible arrangement for the growth in the [110] direction.

Cleaving KBr crystals in vacuum and annealing them at moderate temperatures results in the formation of wide terraces with step edges in [100] direction which can be as long as several hundred nanometers. Evaporating the cyano-porphyrins onto the bulk KBr(001) surface results, as also reported earlier [25–26], in the formation of molecular wires. Figure 1 shows a topographic measurement on a KBr(001) surface, decorated with cyano-porphyrines. The molecular monowires (1) are found to be more than 700 nm long mainly depending on the length of the step. Along one and two monolayer step edges, single molecular wires are found while at higher steps disordered aggregates of molecules (2) appear. Based on simple geometrical considerations and taking into account the strong dipole moment of the molecules, the special expansion of the aryl side groups and the enhanced electrostatic field at the step edge result in a basic model of the wire formation as presented in Figure 1b, Figure 1c and Figure 1d. Single molecules are highly mobile at rt at the surface. Due to an electrostatic interaction between the dipole moments of the molecules and the enhanced periodic electrostatic field at a step edge compared to the flat surface, the molecules are attracted towards the steps. A stabilization of the wire is enabled due to a π–π binding between the porphyrin cores of the molecules. Increasing the step height changes the tilt angle of the molecules towards the surface which is inherently coupled with a weaker π–π interaction and therefore a more fragile molecular wire.

The analysis of our measurements resulted in a wire height of 0.8−0.9 nm and of 1.2−1.3 nm for the one and two monolayer (1ML and 2ML) step edges, respectively. Assuming that the height of a single molecule from the cyanophenyl to the upper parts of the aryl groups is roughly 1.5 nm (the calculated height would be 14.989 Å plus the van der Waals radii of one hydrogen and one nitrogen atom) and that the molecules are not laterally tilted, this would lead to a tilt angle of roughly 57 ± 5° for the 2ML and 35 ± 5° for the 1ML step edge with respect to the substrate. Balaban et al. [37] showed that the distance between two molecules in the π–π plane is approximately 3.6 Å, which leads to a distance of 5.9 Å parallel to the surface for a tilt angle of 37° between the porphyrin core and the π–π direction. This angle is observed in crystallographic assemblies of those molecules as well as in former nc-AFM studies [25–26]. Taking also into account a vertical tilt angle, determined by the aryl groups (Figure 1b and Figure 1c), the measured height of the molecules results in a tilt angle in the π–π stack direction of 37° and 43° for the 1ML and the 2ML step, respectively. Both values indicate a stable π–π interaction, while for 3ML steps and higher no stable configurations can be found for a single molecular wire (see Figure 1d). As already visible in Figure 1a, the unordered agglomerates (3) are the source of multi-wire structures. These structures are parallel ordered single molecular wires growing in the 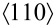 directions on KBr. High-resolution nc-AFM measurements of these structures [26] revealed a separation of the single wires by 2.4 ± 0.2 nm which corresponds to approximately five lattice spacings of the substrate. Pšenčík et al. determined distances between different bacteriochlorophyll stacks of 2.1–3.0 nm in natural chromosomes; the same order of magnitude as observed for the porphyrin assemblies [38]. Since the photon capture crosssection might be markedly increased, hence, leading to higher efficiencies with a broader wavelength range compared to silicon solar cells, such antennae systems are for example also of potential interest for hybrid solar cells that could operate under low or moderate light conditions. Furthermore, porphyrins are known to be very promising building blocks: They are not only very stable, inexpensive and quickly accessible, but also both the periphery and the central metal are very easy to modify. Therefore, such porphyrin wires can be tuned with a high degree of freedom.

**Figure 2:**
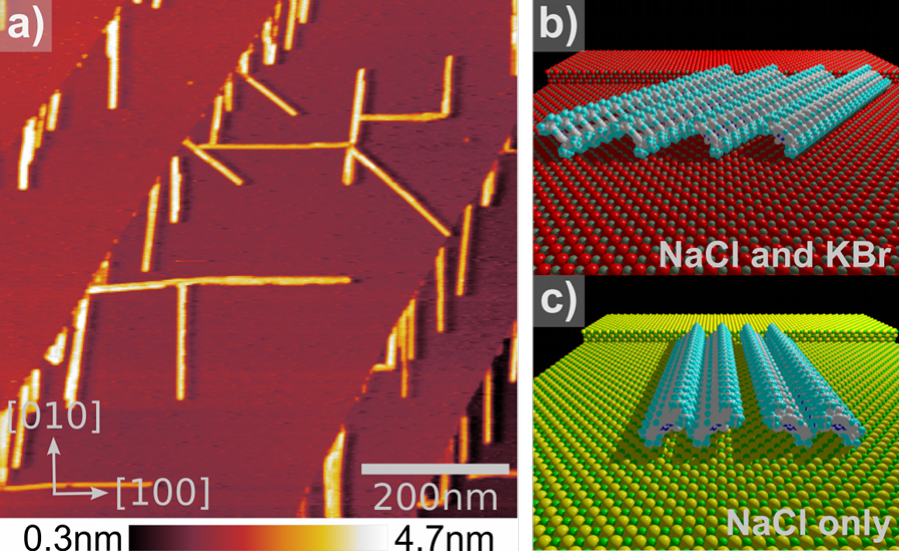
(a) Topography of cyano-porphyrin molecular wires on a NaCl single crystal surface. In contrast to the growth on KBr, the molecular wires are also oriented along the [100] direction of the substrate. In (b) and (c) the two different growth directions are schematically visualized.

FFT-analysis of measurements [26] showing simultaneous molecular and atomic resolution of the substrate revealed that, unlike at step edges in [100] direction, the molecule-to-molecule distance within a wire differs from the KBr lattice spacing. The molecule–molecule separation measures 5.6 Å, compared to the distance of 4.6 Å between two K^+^ ions along the 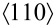 directions. This indicates that the dimensions of the molecule ask for a larger separation than the K^+^ ions intervals could provide, rather corresponding to the spacings observed in the crystal lattice of Balaban et al. [37]. At the steps along the [100] direction, K^+^ ions are alternating with Br^−^ ions creating attractive and repulsive sites for the partially negative charged cyano-groups and therefore forcing them into position. Diagonally across the lattice in 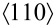 directions, the K^+^ ions are evidently closer together and not interrupted by bromine ions, presumably creating a slightly delocalized positive charge distribution. The stacks are directed along one dimension but in contrast to the assemblies at the step edges, the single porphyrins are not located each directly above a potassium ion, but rather along the K^+^ chain, keeping their thermodynamically preferred intermolecular spacing. The molecular wires are most likely inclined to the surface, with the cyano-groups pointing downwards and the big side groups standing out more on one side (Figure 2b). Heights between 1.5–2.0 nm were measured for multi-wires, depending on the tilt angle of the stacks respective to the surface.

Looking at the spacing of 5.6 Å between the stacked molecules leads to the assumption that NaCl with a lattice constant of 5.65 Å is an ideal substrate to grow multi-wires on. NaCl is chemically and physically similar to KBr and is therefore a good sample to investigate the influence of the lattice distance of the substrate to the self-assemblies. Figure 2a shows an overview nc-AFM image of the molecular assemblies on NaCl(001). The step edges have no specific direction and show no ordered molecular decorations. Regions with steps in 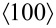 directions show similar single-wire decoration as the KBr(001) surface did. Additionally, we observe a large amount of broader structures growing across the terraces which presumably start growing from kink sites at the step edges. The main difference which was observed between self-assembly on KBr and NaCl is the tendency of the molecules to form crossing carpets or networks of wires. Figure 2a shows such a network of several wire-junctions. The angles between the structures are not only 90° as observed on KBr but also 45° indicating a growth oriented in all major crystallographic surface directions. The structures along the 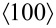 directions do not differ in shape or thickness from the ones oriented in the 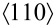 directions. The molecules along the 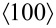 directions on NaCl are most certainly adsorbed at every sodium atom. That leads to an intermolecular distance of 5.65 Å making wire growth along this direction more favorable compared to KBr. However, wires along the 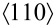 directions can still grow from kink sites or wire junctions. In Figure 2b and Figure 2c structural models for KBr(001) and NaCl(001) are presented.

**Figure 3:**
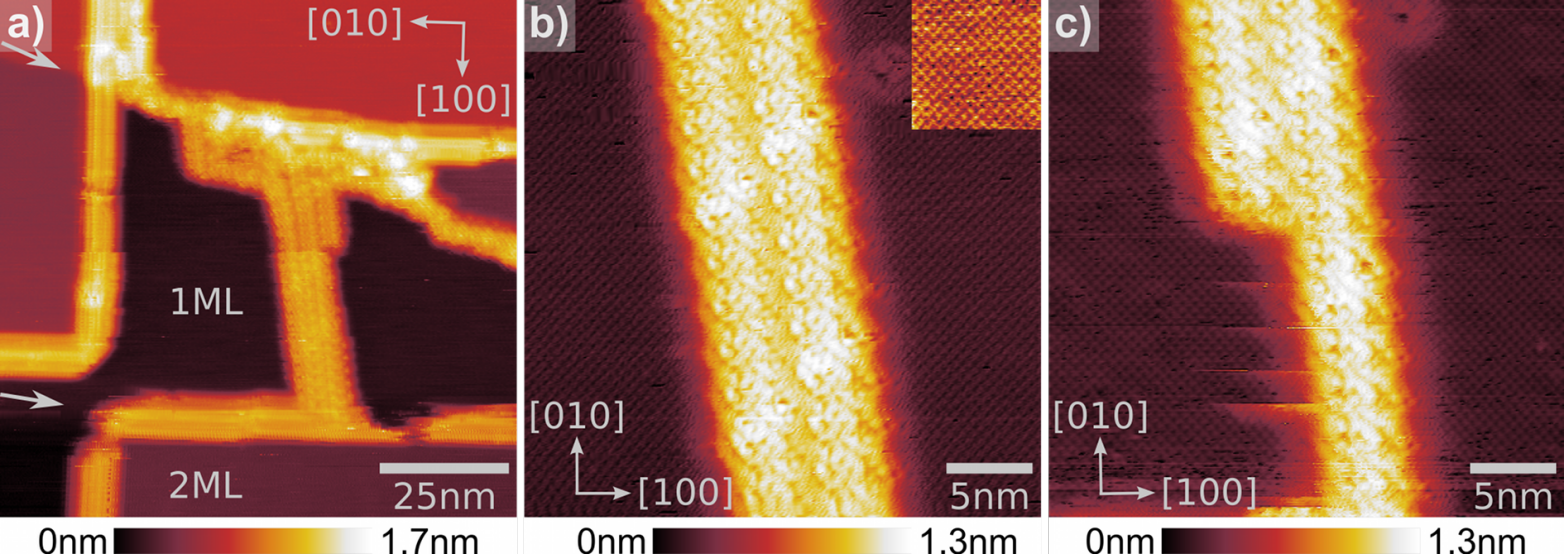
nc-AFM measurements of molecular assemblies grown on an ultrathin KBr layer on Cu(111). (a) 100 × 100 nm^2^ overview of ordered cyano-porphyrin assemblies on single and double KBr layers. (b) and (c) 30 × 30 nm^2^ zoom in of the free standing molecular assembly on a single KBr layer. Clear sub-molecular resolution as well as atomic resolution is observed. After decreasing the set-point, parts of the assembly are removed and the atomic corrugation below becomes visible.

To study the influence of a metal substrate on the formation of the molecular wires and assemblies, we evaporated the cyano-porphyrin molecules onto thin KBr films grown on Cu(111). In Figure 3, a series of topographical images can be seen. In (a) a 100 × 100 nm^2^ overview of ordered cyano-porphyrin assemblies on single and double KBr layers is shown. KBr steps in 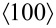 directions of the second ML are decorated by not only one single molecular wire as observed on the bulk material but with a multi-wire. Furthermore, Cu steps indicated by small arrows from the left to the right side of the image overgrown by KBr are also partially decorated by the molecules. Additionally, an assembly is originated from the underlying Cu step and grows towards the lower image edge. It is then interrupted by a conventional wire along a KBr island. Figure 3b and Figure 3c show a 30 × 30 nm^2^ topography image of the assembly, already revealing submolecular details as well as atomic resolution of the underlying KBr. First, the molecular assembly is not aligned along a certain substrate direction of the KBr layer. The rows are inclined by ≈10° to the [010] direction of the KBr layer. Second, submolecular contrast does not reveal a wire like configuration as observed for the multi-wires on bulk ionic crystals. The molecules lay rather flat on the surface since the whole structure is only 0.9–1.0 nm in height.

These measurements also reveal the relatively weak binding energy of the molecules to the substrate: Already during the change of the set point, parts of the layer on the left lower side were removed while scanning from bottom to top. The first few lines of Figure 3c were scanned with an increased frequency shift of Δ*f*_1st_ = −11 Hz. After the removal of the first molecules, the set point was lowered to Δ*f*_1st_ = −10 Hz again. Regardless of that, the tip continued to remove molecules thinning the structure to 50% of its original size. It is remarkable that even though the tip is removing molecules the scan remained absolutely stable and maintained a high resolution ability during all the performed manipulations. The amount of removed molecules and the shape of the resulting structure suggest that the molecules are arranged in a superstructure of about 6–8 nm width. Both columns visible in Figure 3b and Figure 3c show periodic and distinct features proving that they are real submolecular features. Nevertheless, suggesting an appropriate model of the molecular arrangement based only on these measurements has proven to be difficult. However, it can be concluded that the influence of the Cu(111) substrate on the molecular assemblies and wires hinders the formation of mono-and multi-wire cyano-porphyrin assemblies stabilized by an intermolecular π–π interaction.

## Conclusion

The adsorption of cyano-porphyrin molecules was studied for bulk KBr and NaCl samples and resulted in various reproducible assemblies on the surfaces. Especially step edges and kinks of the alkali halide crystals act as trapping points for the polar molecules, preventing them from diffusing freely over the surface. Simultaneously, intermolecular interactions force the cyano-porphyrins to form π–π stacks. These wires grow along the edges, forming long one-dimensional molecular structures. The growth is affected by the potential corrugation at the step edge which forces the negatively charged nitrogen atom of the cyano-porphyrin to sit on top of a positively charged ion. This results in an intermolecular distance corresponding to the lattice constant of the underlying substrate. At increased molecule coverages, two-dimensional arrays start to grow away from the steps across the terraces. The preferred growth orientation is the (110) direction on KBr while on NaCl also assemblies oriented in (100) direction are found. The different growth mode is directed by the lattice spacing of the underlying substrate and the equilibrium distance of the π–π interaction of the molecules. The absorption behavior of the cyano-porphyrins was also studied on ultrathin KBr films on Cu(111). We have shown that KBr thin films can be used as a substrate for the molecular assemblies at room temperature. Nevertheless, the first layers of KBr are still not sufficient to decouple the molecules completely from the underlying Cu substrate. On areas close to an underlying copper step, porphyrins grow in a hexagonal lattice structure and are probably adsorbed with their core more parallel to the surface loosing their intermolecular π–π stacking.

## Experimental

**Figure 4:**
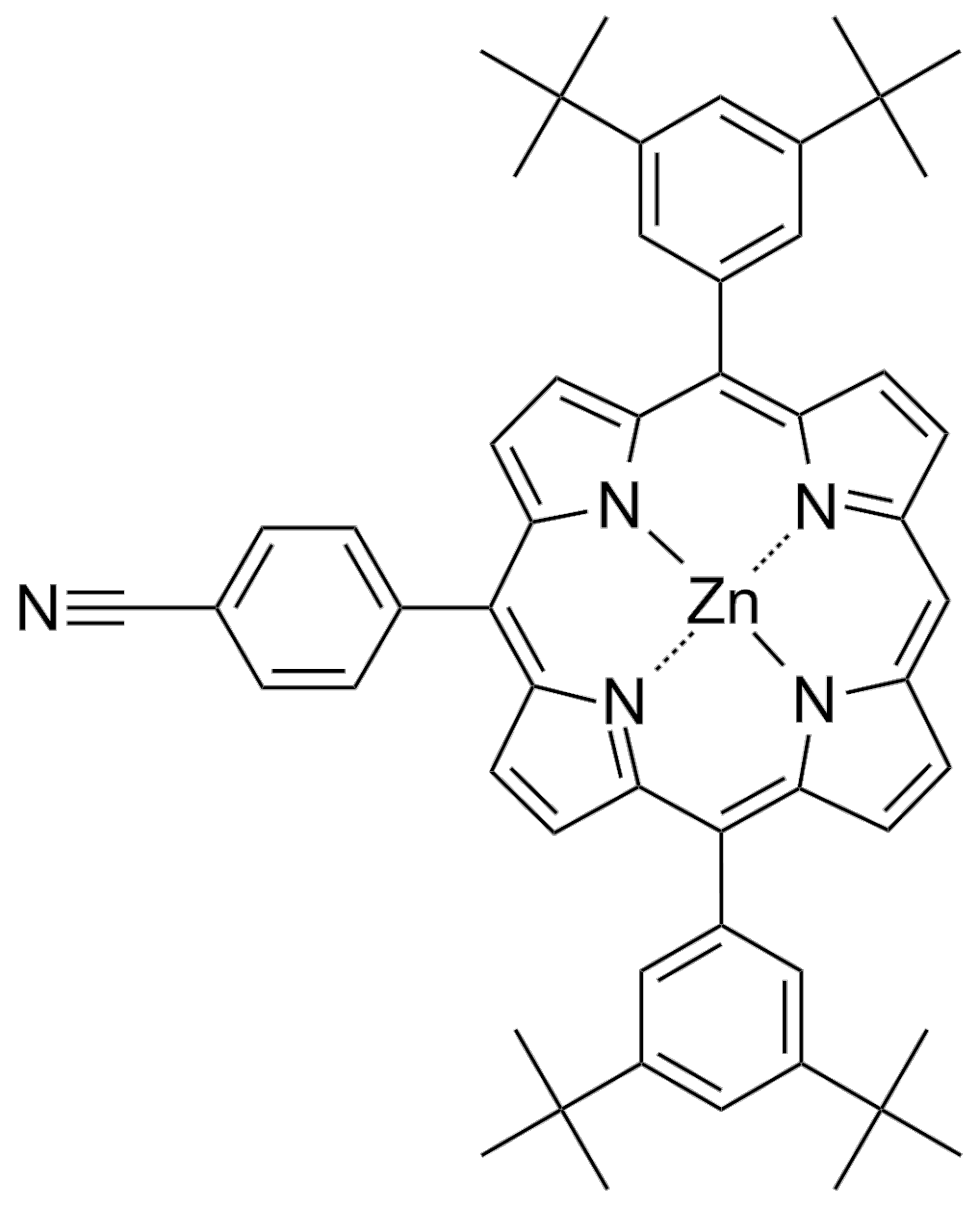
Chemical structure of the *meso*-(4-cyanophenyl)-substituted Zn(II) porphyrin investigated in this study [40]. The dipole moment of the molecule along the C–N bond is 4.37 D.

Experiments were performed under ultrahigh vacuum (UHV) conditions with a base pressure below 10^−10^ mbar using a home built non-contact atomic force microscope operated at rt [39]. In the nc-AFM mode, the tip-sample distance is usually controlled by maintaining a constant shift of the first flexural resonance frequency *f*_1st_ with respect to the resonance far from the surface. Highly doped silicon cantilevers with integrated tips (Nanosensors, NCL), a typical resonance frequency *f*_1st_ ≈ 160 kHz and a spring constant *k* ≈ 30 N/m were employed as a force sensor. The typical oscillation amplitude measures about *A*_1st_ ≈ 5–20 nm. The cantilevers were annealed in UHV (30 min at 120 °C) and sputtered (1–2 min at 680 eV) with Ar^+^ ions for cleaning. In the experiments reported here, *meso*-(4-cyanophenyl)-substituted Zn(II) porphyrin (cyano-porphyrin, Figure 4) was thermally evaporated from a Knudsen cell at 160 °C (with a rate of the order of 10 Å/min) onto bulk crystals of NaCl and KBr as well as on ultrathin KBr layers on a Cu(111) substrate. During evaporation the samples were held at 80 °C to enhance the diffusion of the molecules at the surface. The synthesis of the cyano-porphyrin molecules has been described in detail in [40]. The bulk crystals were cleaved in UHV followed by an annealing step at 150 °C to reduce surface charges. In our experiments we used additionally a Cu(111) surface which was prepared in UHV according to regular surface science techniques by several cycles of Ar^+^ ion bombardment and subsequent annealing to 520 °C. KBr thin films were deposited on the clean Cu(111) substrates by sublimation, using a temperature controlled Knudsen cell. As a source material, crushed salt powder obtained from alkali halide single crystals was used. In order to obtain thin layers of KBr, choosing a very low evaporation rate of ≈0.2 Å/min proved to be successful.
